# Valorization of Fish Processing By-Products: Microstructural, Rheological, Functional, and Properties of Silver Carp Skin Type I Collagen

**DOI:** 10.3390/foods11192985

**Published:** 2022-09-24

**Authors:** Yongxin Guan, Jianlin He, Junde Chen, Yushuang Li, Xingkun Zhang, Yan Zheng, Linyan Jia

**Affiliations:** 1 College of Chemistry and Chemical Engineering, Mudanjiang Normal University, Mudanjiang 157011, China; 2 Technical Innovation Center for Utilization of Marine Biological Resources, Third Institute of Oceanography, Ministry of Natural Resources, Xiamen 361005, China

**Keywords:** silver carp, collagen, by-products, functional properties, rheological properties

## Abstract

The objective of this study was to develop aquatic collagen production from fish processing by-product skin as a possible alternative to terrestrial sources. Silver carp skin collagen (SCSC) was isolated and identified as type I collagen, and LC-MS/MS analysis confirmed the SCSC as *Hypophthalmichthys molitrix* type I collagen, where the yield of SCSC was 40.35 ± 0.63% (dry basis weight). The thermal denaturation temperature (T_d_) value of SCSC was 30.37 °C, which was superior to the collagen of deep-sea fish and freshwater fish. Notably, SCSC had higher thermal stability than human placental collagen, and the rheological experiments showed that the SCSC was a shear-thinning pseudoplastic fluid. Moreover, SCSC was functionally superior to some other collagens from terrestrial sources, such as sheep, chicken cartilage, and pig skin collagen. Additionally, SCSC could provide a suitable environment for MC3T3-E1 cell growth and maintain normal cellular morphology. These results indicated that SCSC could be used for further applications in food, cosmetics, and biomedical fields.

## 1. Introduction

Collagen is the main component of the extracellular matrix of animals and is found in bones, skin, and tendons, accounting for about 30% of the total protein content. Collagen consists of three polypeptide chains twisted to create a triple helix structure, providing mechanical stability, flexibility, and strength for the organism [1]. Scholars have identified 29 different types of collagens with different distinct amino acid sequences and molecular structures [2]. Among the discovered collagens, type I collagen has a wide range of food, cosmetic, and biomedical applications due to its excellent biocompatibility, low antigenicity, and high biodegradability [3].

Traditional type I collagen found on the market is primarily derived from the skin and bones of mammals such as cattle and pigs. In recent years, outbreaks of terrestrial infectious diseases such as foot and mouth disease, avian influenza, and Creutzfeldt–Jakob disease have caused restrictions on collagen applications. Religious factors also limit the use of collagen of terrestrial origin [4]. The currently available sources of collagen cannot satisfy the increasing consumer demand, and new sources of collagen are urgently required. Marine collagen has attracted the attention of researchers. Nalinanon et al. (2010) [5] extracted collagen from the Arabesque greenling skin and examined its properties. Sun et al. (2017) [3] extracted Pacific cod skin collagen and evaluated its biocompatibility. Li et al. (2013) [6] extracted collagen using two different methods to extract collagen from the skin and bones of Spanish mackerel. Although fish skin collagen research has focused on marine fish, the poor thermal stability of deep-sea fish collagen has limited its application. Freshwater fish live in a temperature environment very different from deep-sea fish, which may result in the better thermal stability of freshwater fish collagen compared to deep-sea fish collagen.

Silver carp (*Hypophthalmichthys molitrix*) is a filter-feeding freshwater fish of the carp genus. It is widely distributed in the principal waters of China and other Asian countries, and is the primary economic fish species in China, with an annual production value of more than 3.8 million tons. About 80% of the by-products from silver carp are not rationally utilized during processing [7]. Some of these by-products are processed into low-value products such as fertilizers, with most discarded in water bodies. The presence of NO_3_^−^ and PO4^3−^ in by-product skins will alter the dissolved oxygen capacity of water, causing eutrophication of the water bodies and disrupting the ecological balance of aquatic organisms [8]. These by-product fish skins contain 50–70% collagen. Thus, the use of silver carp skin may be an effective way to obtain high-value-added products as an alternative source of traditional collagen. In recent years, researchers have obtained collagen from the skin and scales of silver carp processed by-products and investigated its structural properties. Zhang et al. (2010) identified the potential of collagen from silver carp skin and scales in food processing [9]. Faralizadeh et al. (2021) determined the feasibility of collagen-based biomaterials from the perspective of cytocompatibility [10]. Sionkowska et al. (2020) analyzed the influence of UV light on rheological properties of collagen extracted from silver carp skin [11]. However, there are few reports that systematically study the rheological and functional properties of collagen from silver carp skin. Rheological properties are crucial parameters required for protein product production. In the processing of collagen products, rheological properties will affect the material properties, and the functional properties are also critical indicators for determining collagen applications.

Therefore, the purpose of this investigation was to address the extraction of silver carp skin collagen (SCSC), and characterize its microstructural, rheological, functional, and biological properties to determine its potential as an alternative source of terrestrial collagen.

## 2. Materials and Methods

### 2.1. Materials

Rat tail type I collagen and protein marker (26634) were purchased from the Sigma Chemical Company (St. Louis, MO, USA), while sodium dodecyl sulfate (SDS), *N, N, N, N*-tetramethylethylenediamime (TEMED), and Coomassie Brilliant Blue R-250 were purchased from Bio-Rad Laboratories (Harkles, CA, USA). MC3T3-E1 cell lines (Cat No. CBP60946) were purchased from Cobioer (Nanjing, China). Other reagents used in the experimental process were of analytical-grade purity.

### 2.2. Preparation of SCSC

SCSC was isolated according to a previously described method with slight modifications [12]. Silver carp (2 years of age, 25–50 cm, 1500–2000 g) was purchased from Fujian Fengsheng Food Co., Ltd. (Zhangzhou, China). Silver carp body skins were manually removed using a scalpel and washed with cold water. These skins were incubated in 150 L of 0.1 mol/L sodium bicarbonate for 3 h with continuous stirring, and then rinsed with cold water until a neutral pH was reached. The resulting material was soaked in 0.5 M acetic acid at 1:40 (*w*/*v*) and stirred for 10 h at 4 °C in a top-mounted electronic stirrer (EUROSTAR 20 digital, IKA, Wilmington Germany). Then, these skins were centrifuged at 9000 rpm at 4 °C for 30 min (Avanti J-26 XP, Beckman, Brea, CA, USA). NaCl was added to the supernatant after centrifugation to achieve a final concentration of 3% (*w*/*v*) and then salted for 30 min. The sediment was collected after centrifugation at 9000 rpm for 30 min at 4 °C, and the precipitate was redissolved in 0.5 M acetic acid at 1:10 (*w*/*v*). After allowing for sufficient dissolution, the solution was placed in dialysis bags (MD 77 MM, Viskase, Lombard, IL, USA) in 0.1 M acetic acid for 24 h, followed by distilled water for 48 h. Then, the dialyzed samples were freeze-dried (Telstar, lyoobeta-25, Barcelona, Spain), and the obtained samples were stored at −20 °C. The yield can be calculated as follows:

(1) $$\mathrm{Yield}\;\left(\%\right)=\frac{M}{M_{0}}\times 100\%,$$

where *M*_0_ is the dry weight of the fish skin (g) and *M* is the weight of the freeze-dried collagen (g).

### 2.3. Sodium Dodecyl Sulfate–Polyacrylamide Gel Electrophoresis (SDS-PAGE)

Collagen samples were mixed with the sample buffer at a rate of 4:1 (*v*/*v*), and the acquired mixture was heated at 100 °C for 3 min. Subsequently, the samples were loaded on 8% separating gel and 3% stacking gel. Electrophoresis gel plates were prepared and electrophoresed at 110 V and 50 mA for 80 min using a mini protein vertical plate electrophoresis system (Bio-Rad Laboratories, Hercules, CA, USA). Electrophoresis was performed after staining the gels using 0.1% (*w/v*) Coomassie Brilliant Blue R-250, 30% (*v*/*v*) CH_3_OH, and 10% (*v*/*v*) acetic acid, and finally the gels were decolorized with 50% (*v*/*v*) C_2_H_5_OH and 10% (*v*/*v*) acetic acid. Rat tail type I collagen was utilized as the standard. The protein molecular weights were estimated using protein markers (26634) and Quantity One 4.6.0 (Bio-Rad Laboratories, Hercules, CA, USA). The band intensities were analyzed using ImageJ software (VERSION 1.8.0, National Institute of Mental Health, Bethesda, MD, USA).

### 2.4. Amino Acid Sequence Analysis

The amino acid sequence determination of SCSC was performed according to the method previously reported [13]. The samples were first subjected to electrophoresis experiments. The SDS-PAGE strips were cut, rinsed with double distilled water, and then decolorized. Subsequently, 0.01 µg/µL of proteomics grade trypsin to obtain the appropriate amount, and then 25 mmol/L of NH_4_HCO_3_ solution containing 10% ACN overlay, in a 37 °C water bath. After digestion, the supernatant was transferred to a new centrifuge tube, and 50 µL of extract was added to the remaining gel, which was centrifuged for 15 min after the water bath. Then, the supernatant was combined, concentrated, and centrifuged, and then dried for mass spectrometry analysis.

The lyophilized peptide samples were redissolved in mobile phase A (0.1% formic acid, 5% acetonitrile/water) with a 2 µL/min C18 pre-column (100 µm × 3 cm, C18, 3 µm, 150 Å). Then they were held on an Easy-nLC1200 liquid phase system (HPLC-MS/MS, Ultimate 3000-API 4000 Q TRAP, Thermo Fisher Scientific, Dreieich, Germany) for 8 min to rinse and desalt the samples. The column size was a C18 reversed-phase column (75 μm × 15 cm C18, 3 μm, 120 Å, ChromXP Eksigent, San Francisco, CA, USA) and the gradient used for experimentation consisted of 10 min LCMS with mobile phase B (0.1% formic acid, 95% acetonitrile/water), increasing from 5% to 40%. Mass spectrometry was performed using a QEactive system, combined with a nanoliter spray III ion source with a spray voltage of 2.3 kV, an air curtain pressure of 30 PSI, a nebulization air pressure of 14 PSI, and a heating temperature of 150 °C. The mass spectrometry scan mode consisted of information-dependent acquisition in IDA, information-dependent analysis mode, with a scan time of 250 ms for a single spectrum of TOF-MS. Up to 26 secondary spectra with 2^+^ to 5^+^ charges and single counts greater than 200 cps were acquired in each IDA cycle, with a cumulative time of 80 ms for each secondary spectrum. The database was NCBI Mascot, the enzyme was trypsin, the maximum allowed missed cut site was 1, the fixed modification was carbamidomethyl (C), the variable modifications were acetylation (protein N-terminal), deamidation (NQ), and Gln->pyro-Glu (N-terminal Q), Glu->pyro-Glu Glu->pyro-Glu (N-terminal E) and oxidation (M), the MS tolerance was 20 ppm, and the MS/MS tolerance was 0.05 Da. Protein confidence above 95% was considered successful for identification.

### 2.5. Spectral Analysis

#### 2.5.1. Ultraviolet Absorption (UV)

Collagen samples were dissolved in 0.5 M acetic acid at a concentration of 1 mg/mL, and the UV absorption value of the sample solution was calculated using a UV-Vis absorption spectrometer (UV-2550 SHIMADZU, Kyoto, Japan) in the wavelength range of 190–600 nm, with 0.5 M HAC as the blank control.

#### 2.5.2. Fourier Transform Infrared (FTIR)

The collagen and KBr mixtures at 1:100 (*w*/*w*) were pressed on a translucent sample sheet and analyzed using an FTIR spectrometer (VERTEX 70, Bruker, Ettlingen, Germany). The scan range was from 400 to 4000 cm^−1^ with a resolution of 4 cm^−1^, using KBr as the blank control.

#### 2.5.3. Circular Dichroism (CD)

Collagen samples were dissolved separately in 0.5 M acetic acid to produce 0.1 mg/mL solutions of collagen. Then, the solutions were centrifuged at 18,000 rpm for 10 min at 4 °C. The supernatants were collected and placed in a quartz cell and the CD spectra were measured on a Chirascon CD spectrometer (Applied Photophysics Ltd., London, UK). The spectra were recorded at 260–190 nm wavelengths at 15 °C in 0.1 nm steps with a response time of 1 s.

#### 2.5.4. X-ray Diffraction (XRD)

Collagen samples were measured using an X-ray diffractometer (X’Pert Pro XRD, PANalytical, Almelo, The Netherlands) with CuKa rays, *λ* = 0.154 nm, a scanning range 0–80°, and an angular velocity 0.06 °/s. The d value was calculated from the Bragg equation, Equation (2):

(2) $$d\left(Å\right)=\frac{\lambda }{2sin\theta },$$

where *λ* is the X-ray wavelength (1.54°) and *θ* is the Bragg diffraction angle.

### 2.6. Determination of Denaturation Temperature (T_d_)

The T_d_ was according to the method published with slight modifications [12]. Collagen was dissolved in 0.5 M acetic acid to 20 mg/mL, and the Td was measured using a rheometer (MCR 302, Anton Paar, Graz, Austria) with a stainless-steel cone/plate geometry (CP 25-2) (2° cone angle, 25 mm cone diameter). The samples were placed in the gap between the top and bottom plates, which were set at 103 μm. Dimethylsilicone oil was applied around the top and bottom plates to avoid evaporation of moisture from the sample. The samples were measured at temperatures from 10 to 50 °C, where the shear rate was constant at 1 s^−1^ and the temperature was elevated at a rate of 3 °C/min. The temperature at which the relative viscosity was 50% was recorded as the T_d_ of the samples, which was calculated according to Equation (3):

(3) $$T_{d}=\frac{A}{B}\times 100\%,$$

where A is the sample viscosity (Pa.s) and B is the sample initial viscosity.

### 2.7. Rheological Properties

The rheological properties of the collagen were examined by dissolving collagen in acetic acid to 5, 10, 15, 20, and 25 mg/mL at 20 °C. The dynamic frequency sweeps were performed for the 15 mg/mL collagen at different temperatures of 20, 25, 30, 35, and 40 °C, while the G′, G”, η*, and tangent angle were determined with a rheometer (MCR 302, Anton Paar, Graz, Austria) using a stainless-steel cone/plate geometry (CP 60-0.5) (0.5° cone angle, 60 mm cone diameter) with a dynamic frequency sweep from 0.01 to 10 Hz in the oscillatory mode. The sample was placed in the gap between the top and bottom plates, which was set at 57 μm. Dimethylsilicone oil was applied around the top and bottom plates to avoid evaporation of moisture from the sample.

### 2.8. Zeta Potential

The Zeta potential was determined according to the method published with slight modifications [14]. The collagen was prepared with 0.1 M acetic acid to 0.2 mg/mL for each 20 mL sample solution. The pH was adjusted to a range of 2–10, with 1.0 M HNO_3_ and 4.0 M NaOH. These solutions were evaluated with a Zeta potential analyzer (Zetasizer Nano ZS90, Malvern Instr., Melvin, UK).

### 2.9. Functional Properties

#### 2.9.1. Foaming Properties

The foaming properties were determined according to the method published with slight modifications [15]. The collagen was formulated with 0.5 M acetic acid to 0.5 (*w/v*). The pH values were adjusted to 2, 4, 6, 6.98, 8, and 10 with 1 M HCl and 4 M NaOH, respectively. Then, the samples were homogenized at 22,000 rpm (JS25, JUNRUI, Yangzhou, China) for 2 min at room temperature, and the foam volumes at 0 min and 1 h were recorded. The foaming capacity (FC) and foam stability (FS) were calculated according to Equations (4) and (5):

(4) $$\mathrm{FC}\%=\frac{V_{2}}{V_{1}}\times 100\%,$$

(5) $$\mathrm{FS}\left(\%\right)=\frac{V_{3}}{V_{2}}\times 100\%,$$

where *V*_1_ is the initial volume (mL), *V*_2_ is the foam volume (mL), and *V*_3_ is the foam volume after 1 h of rest (mL).

#### 2.9.2. Emulsifying Properties

The emulsifying properties were determined according to the method published with slight modifications [15]. The collagen was formulated with 0.5 M acetic acid to 0.5% (*w/v*), and the pH was adjusted to 2, 4, 6, 6.98, 8, and 10 with 1M HCl and 4 M NaOH. The samples were incorporated with 5 mL of corn oil and homogenized at 22,000 rpm (JS25, JUNRUI, Yangzhou, China) for 1 min. The emulsions were diluted with 0.1% SDS for 0 and 10 min. Then, the oscillator was shaken for 30 s and 0.1% SDS was used as the blank control to evaluate the absorbance at 500 nm. (UV-2550 SHIMADZU, Kyoto, Japan). The emulsification activity index (ESI) and the emulsion stability index (EAI) were calculated according to Equations (6) and (7):

(6) $$\mathrm{EAI}\left(\frac{m^{2}}{g}\right)=\frac{2\times 2.303\times A_{0}}{\phi \times weight\;of\;protein}\;,$$

(7) $$\mathrm{ESI}\left(\min\right)=\frac{A_{10}\times \Delta t}{\Delta A},$$

where *A*_0_ is the absorbance at 0 min, *A*_10_ is the absorbance at 10 min, Δ*A* is *A*_10_–*A*_0_, Δ*t* is 10 min, and *Φ* is the oil volume fraction.

#### 2.9.3. Water Absorption Capacity

The water absorption capacity (WAC) was determined according to the method published with slight modifications [16]. Collagen (0.2 g) was combined with 20 mL of distilled water and then vortexed for 30 s using a vortex mixer (MX-E, SCILOGEX, Beijing China). Then, the samples were allowed to settle at room temperature for 60 min and were centrifuged at 6000 rpm/min for 10 min at 4 °C. The supernatant was removed and the aspirated collagen was weighed. The water absorption was calculated according to Equation (8):

(8) $$\mathrm{WAC}\left(\frac{g}{g}\right)=\frac{W_{2}-W_{1}}{W_{0}},$$

where *W*_0_ is the collagen weight, *W*_1_ is the total mass of the sample and the tube, and *W*_2_ is the total mass of the sample and tube after centrifugation.

#### 2.9.4. Oil Absorption Capacity 

The oil absorption capacity (OAC) was calculated according to the published method with slight modifications [16]. Collagen (0.2 g) was combined with 5 mL of corn oil and then vortexed for 30 s using a vortex mixer (MX-E, SCILOGEX, Beijing, China). The samples were allowed to settle at room temperature for 60 min and then were centrifuged at 6000 rpm/min for 10 min at 4 °C. The supernatant was removed, and the aspirated collagen was weighed. The oil absorption was calculated according to Equation (9):

(9) $$\mathrm{OAC}\left(\frac{g}{g}\right)=\frac{F_{2}-F_{1}}{F_{0}},$$

where *F*_0_ is the collagen weight, *F*_1_ is the total mass of the sample and tube, and *F*_2_ is the total mass of the sample and tube after centrifugation.

### 2.10. Biological Properties

The biological properties of collagen on the MC3T3-E1 cells were estimated using a CCK-8 assay with some modifications, according to the method published [10]. The collagen was dissolved in distilled water to 0.5 mg/mL. The bottoms of 48-well plates were coated with the collagen solution and dried under a laminar flow hood before they were sterilized with UV light. The cells were inoculated at a density of 1 × 10^4^ cells per well and then incubated at 37 °C in a humidified atmosphere containing 5% CO_2_ for 1, 3, and 5 days. Then, CCK-8 solution was separately added to each sample and incubated for 1.5 h. The absorbance of the samples was measured using a multimode reader (Mithras^2^ LB 943, Berthold, Germany) at a wavelength of 450 nm. The cell viability was calculated according to Equation (10):

(10) $$\mathrm{Cell}\;\mathrm{Viability}\%=\frac{A_{2}}{A_{1}}\times 100\%,$$

where *A*_1_ is the absorbance of control; *A*_2_ is the absorbance of treatment.

### 2.11. Statistical Analysis

All experiments were performed in triplicate and the results were expressed as the mean ± standard deviation (SD). All data were analyzed by ANOVA using SPSS version 17.0 software (IBM SPSS Statistics, Ehningen, Germany), with values of *p* < 0.05 indicating significant deviation.

## 3. Results and Discussion

### 3.1. Yield

The yield of SCSC was 40.35 ± 0.63% (dry basis weight), while the SCSC yields were higher than the bigeye tuna 16.7 ± 0.7%, (dry basis weight) [2], large fin long barbel catfish 16.8% (dry basis weight) [17], and loach 22.42% (dry basis weight) [18]. The difference in yield was possibly due to the different degrees of cross-linking of the collagenous protofibrils in the different fish raw materials [12]. The degree of cross-linking in the terminal peptide region of the collagen molecule is an essential factor in its yield. The low degree of cross-linking of collagen leads to decreased intermolecular forces of collagen molecules. The decreased intermolecular forces of collagen molecules lead to the higher solubility of collagen, thus, providing a higher collagen yield [2]. Additionally, as silver carp aquaculture is widespread in China, with an annual production value of more than 3.8 million tons, these values indicated that silver carp may provide sufficient skin raw materials for collagen production, and SCSC may have the potential to replace collagen in the market.

### 3.2. SDS-PAGE

**Figure 1:**
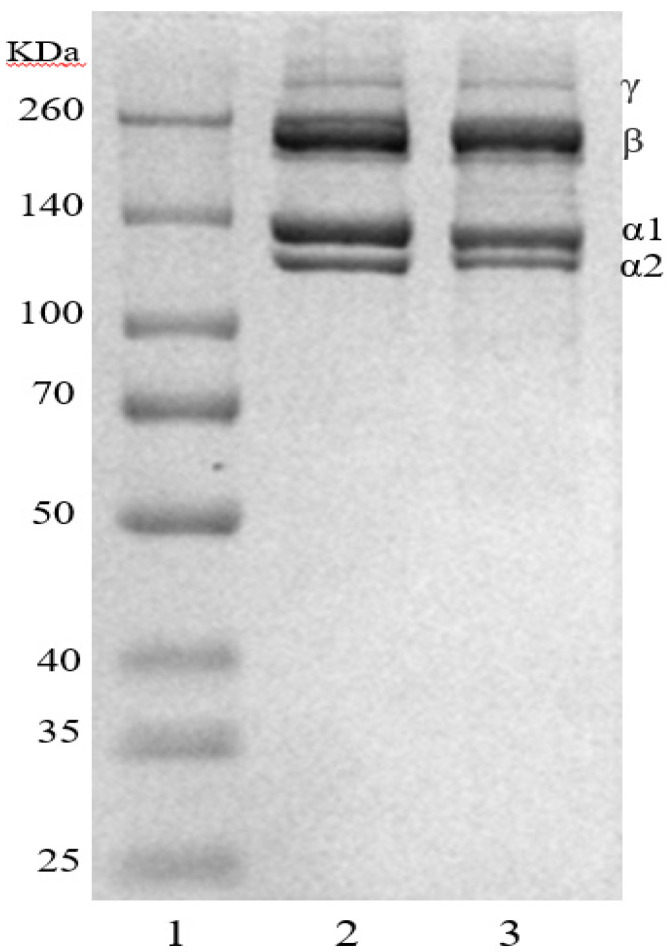
SDS-PAGE pattern of SCSC. (1) Marker; (2) rat tail collagen; (3) SCSC.

The protein patterns from the SCSC and rat tail type I collagen are shown in Figure 1, which all consisted of two distinct α chains (α_1_ and α_2_ chains) and their intramolecular cross-linked dimers (β-chains). The molecular weights of SCSC (α_1_ 129 kDa, α_2_ 121 kDa, and β 224 kDa) were slightly lower than rat tail collagen (α_1_ 132 kDa, α_2_ 121 kDa, and β 230 kDa). These differences were possibly caused by the different collagen sources of mammals and aquatic animals [19]. The clear electrophoretic bands of SCSC and the absence of spurious bands also suggested a higher purity of the extracted collagen. In addition, the gray ratio between the α_1_ and α_2_ chains of SCSC was 2.07:1, which was close to 2:1. This was consistent with the composition of [(α_1_)_2_α_2_], which was similar to previously published collagen derived from skipjack tuna (2:1) and pufferfish (approx. 2:1), indicating that the prepared collagen was type I collagen. [20,21]. 

### 3.3. Amino Acid Sequence Analysis

The amino acid sequences determined using the LC-MS/MS analysis results are presented in the Supplementary Materials. The type of fish skin collagen was determined by ion mass matching of the collagen subunit MS/MS fragments, amino acid sequence coverage, and the fractions calculated from the number of matching peptides of the collagen subunit peptides in the NCBI database.

Among the data detected for α_1_, five proteins were observed with relatively high scores, with a high number of significant matches. First, the GI was AIL02135.1, the score was 847, the mass was 138,365, the sequence was 19, the coverage was 20%, and the protein species was *Hypophthalmichthys molitrix* with collagen type I alpha 1. Second, GI was AGH32451.1, the fraction was 164, the mass was 42,295, the sequence was 4, and the protein species was *Hypophthalmichthys molitrix* actin alpha 1b. Third, the GI was AUF74474.1, the fraction was 158, the mass was 127,755, the sequence was 5, and the protein species *Hypophthalmichthys molitrix* collagen type I alpha 2. Fourth, the GI was AIE40058.1, the fraction was 50, the mass was 13,041, the sequence was 1, and the protein species was *Hypophthalmichthys molitrix* histone H2A, partial. Fifth, the GI was ACO51127.1, the fraction was 33, the mass was 7835, the sequence was 1, and the protein species was *Hypophthalmichthys nobilis* bactin1 protein, partial.

The identical α_2_ also obtained five sets of data with high confidence, as shown in Table 1. The first GI was AUF74474.1, with a score of 2009, the mass was 127,755, the sequence was 31, the coverage was 26%, and the protein species was *Hypophthalmichthys molitrix* collagen type I alpha 2. Second, the GI was AGH32451.1, the score was 121, the mass was 42,295, the sequence was 4, and the protein species was *Hypophthalmichthys molitrix* actin alpha 1b. Third, the GI was AHY86406.1, the score was 76, the mass was 55,200, the sequence was 1, and the protein species was *Hypophthalmichthys nobilis* ATP synthase, H+ transport, mitochondrial F1 complex, beta subunit. Fourth, the GI was AIL02135.1, the score was 39, the mass was 138,365, the sequence was 2, and the protein species was *Hypophthalmichthys molitrix* collagen type I alpha 1. Fifth, the GI was ACO51127.1, the score was 33, the mass was 7835, the sequence was 1, and the protein species was *Hypophthalmichthys nobilis* bactin1 protein, partial.

The results presented above showed that the extracted collagen had the highest coverage of silver carp type I α_1_ and the highest coverage of silver carp type I α_2_ (α_1_: 20% α_2_: 26%), where the relative molecular masses (α_1_: 138,365 Da, α_2_: 127,755 Da) were similar to the SDS-PAGE results (α_1_: 129,000 Da, α_2_: 121,000 Da). This indicated that the experimentally extracted collagen was a typical type I *Hypophthalmichthys molitrix* collagen.

### 3.4. Spectral Analysis

The structure of SCSC was identified using UV absorption spectra, FTIR, CD, and XRD spectroscopy.

**Figure 2:**
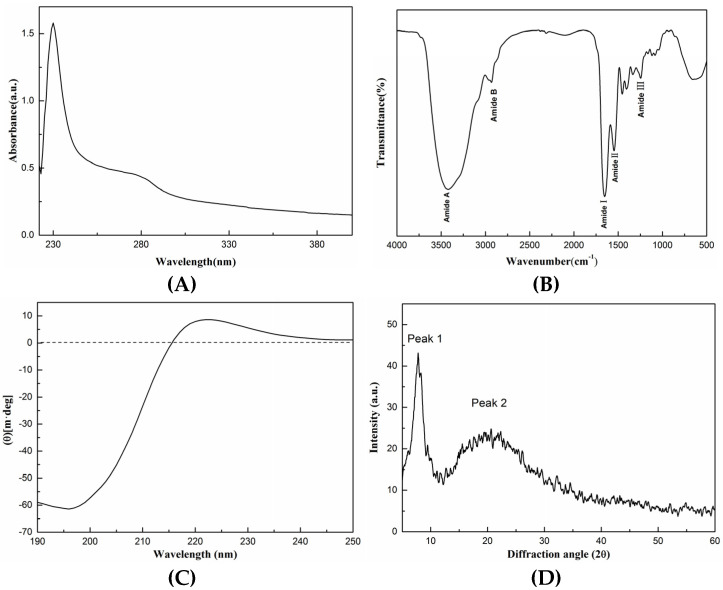
Spectral analysis of SCSC. (**A**) UV; (**B**) FTIR; (**C**) CD; (**D**) XRD.

As shown in Figure 2A, the UV spectrum of SCSC produced two characteristic absorption peaks, where the maximum appeared at 230 nm. The maximum absorption peak was due to the fact that collagen contains C=O, COOH which was a chromophore and the unsaturated group leaps after the absorption of light radiation [22]. In addition, SCSC contained small amounts of tyrosine, phenylalanine, and tryptophan, which consisted of aromatic amino acids with conjugated double bonds in the phenyl-producing peaks with low absorption at 280 nm. The results of this assay were comparable to hammerhead shark type I collagen and puffer fish type I collagen, showing that the extracted collagen was classic type I collagen [23,24]. 

As shown in Figure 2B, the FTIR spectrum of SCSC was similar to that of type I collagen with five absorption peaks including Amide A, Amide B, Amide I, Amide II, and Amide III. The Amide A band of SCSC was observed at 3419 cm^−1^, which was related to the N-H stretching vibrations. According to the study, the N-H stretching vibrations ranged from 3400 to 3440 cm^−1^, when the N-H group was bonded to hydrogen the position of Amide A shifted to a much lower frequency [25]. The Amide B band of SCSC was observed at 2931 cm^−1^, which was related to the asymmetric stretching of CH_2_, which usually occurred at 2900–2950 cm^−1^ [26]. The absorption peaks of Amide I, Amide II, and Amide III bands appeared in the ranges of 1600–1700, 1550–1600, and 1235–1240 cm^−1^, respectively, and their production was related to the C=O, N-H, and C-N stretching vibrations, respectively [6]. The absorption peaks of the Amide I, Amide II, and Amide III bands of SCSC were produced at 1652, 1546, and 1245 cm^−1^. In addition, the absorbance ratio of 1.1 between Amide III and 1400–1454 cm^−1^ was approximated to be 1.0, which indicated that the triple helix structure of collagen was preserved intact [27]. The results of this experiment were similar to the results previously reported for Spanish mackerel and sea cucumber [6,28]. 

As shown in Figure 2C, the CD of SCSC produced two characteristic absorption peaks that appeared at 196 and 222 nm, respectively. This produced a negative peak at 196 nm and a positive peak at 222 nm with an ellipticity of 0 near 215 nm, which was similar to sliver scale lizardfish scale and pufferfish skin. In addition, the Rpn value of the sample was 0.14, and the study showed that the Rpn value was between 0.12 and 0.15, proving that the triple helix structure of the collagen sample remained intact [18]. The results indicated that SCSC had an intact triple helix structure.

As shown in Figure 2D, the XRD spectra of SCSC produced two characteristic absorption peaks. The first sharp peak appeared near 7.46°. This diffraction peak was produced by the distance between the molecular chains of the collagen fibers [29]. The second broad peak appeared near 20.7° and this peak arose from diffuse scattering caused by the many structural layers of the collagen fibers [30]. The XRD results were evaluated according to the Bragg equation. The distances between the molecular chains of the collagen fibrils d_1_ = 1.18 nm and collagen skeleton d_2_ = 0.43 nm were calculated, indicating that the d values were similar to type I collagen diameter, proving the presence of an intact triple helix structure in the samples [12].

### 3.5. Determination of Denaturation Temperature (T_d_)

**Figure 3:**
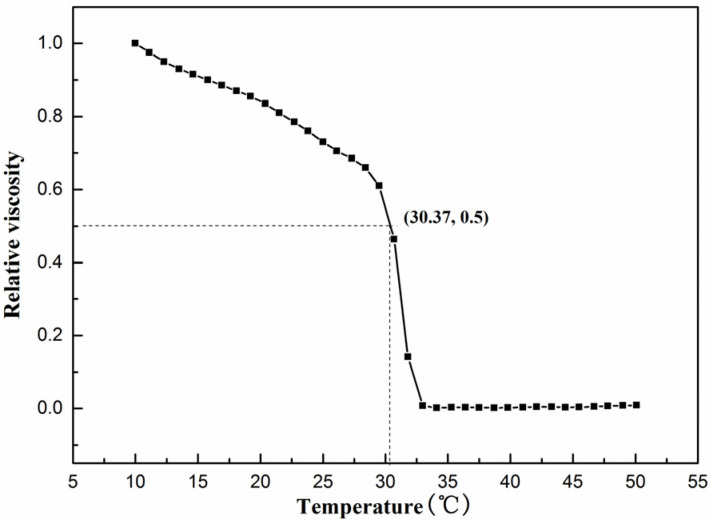
Thermal denaturation curve of SCSC.

Thermal stability is considered one of the most critical properties of collagen. As the temperature increased, the hydrogen bonds in the collagen molecules were destroyed and the triple helix structure dissociated. This modification of the structure weakened the molecular forces between collagen, resulting in a decrease in viscosity. The temperature corresponding to the viscosity shift to half of the original was known as T_d_. As shown in Figure 3, the viscosity of the sample gradually decreased with increasing temperature, becoming half of the initial viscosity at 30.37 °C. After continued temperature increase, the viscosity dropped to a minimum, indicating that the T_d_ of SCSC was 30.37 °C. At this moment, SCSC absorbed enough energy from the outside world and the non-covalent bonds that held the triple helix structure inside rupture. The triple-stranded helical conformation of collagen subsequently converted to an irregular convoluted conformation, and this change caused the physical properties of collagen to change with an increase in temperature [31]. 

In addition, the living environment can lead to differences in T_d_. SCSC showed higher thermal stability than the collagen from deep-sea fish such as Sharpnose skin (28 °C), Pacific cod skin (14.5 °C), and Arabian mackerel (15.5 °C) [1,3,5]. This was possibly due to the fact that deep-sea fish are typically found in environments of high salinity, high pressure, and low temperature. In addition, T_d_ of SCSC was better than some freshwater fish with similar survival environments, such as sturgeon at 26.86 °C and Mozambique tilapia at 28 °C [32]. In general, the T_d_ value of fish collagen was lower than that of mammals, surprisingly, and the T_d_ of SCSC was higher than that of human placental collagen (28.5 °C) [33]. The higher Td made SCSC less susceptible to denaturation during processing at room temperature and it could maintain a stable triple helix structure. This meant that its biological properties (low antigenicity, biocompatibility) remain unchanged. These factors indicated that SCSC might have the potential to be used in food and biological functional material.

### 3.6. Rheological Properties

**Figure 4:**
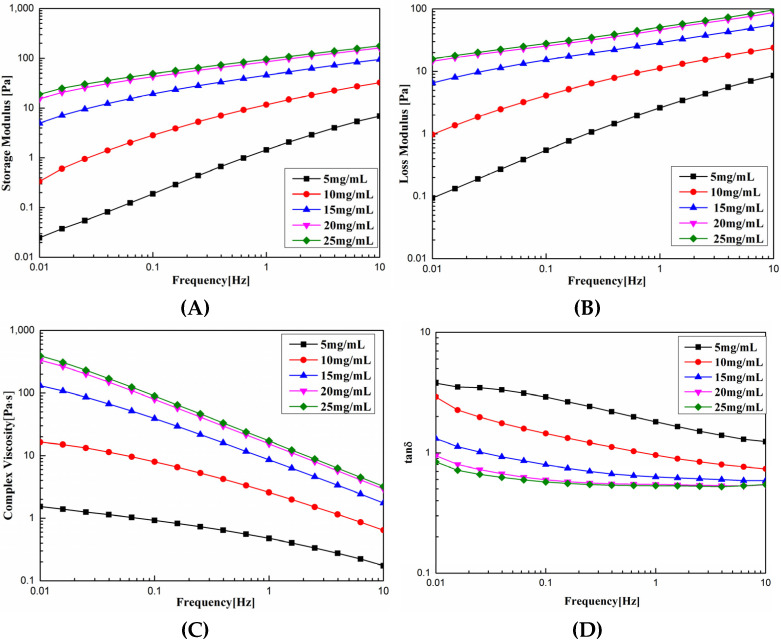
Dynamic rheological curve of SCSC at different concentrations. (**A**) Storage modulus (G′); (**B**) loss modulus (G”); (**C**) complex viscosity (η*); (**D**) tanδ.

As shown in Figure 4A,B, the G′ and G” of SCSC were proportional to the concentration at 0.01 Hz, with the concentration increasing from 5 to 25 mg. G′ increased from 0.0248 to 18.9 Pa, increasing by three magnitudes, and G” increased from 0.0937 to 15.9 Pa, increasing by three magnitudes. At 10 Hz, G′ and G” increased from 6.88 and 8.52 Pa to 177 and 96.3 Pa, respectively, for an increase in three magnitudes and two magnitudes, respectively. These results indicated that the effect of concentration on the solution viscoelasticity was more significant at low frequencies. As shown in Figure 4C η* increased linearly with concentration, when the viscosity of 5 mg SCSC was 1.54 Pa.s. The viscosity of 25 mg SCSC reached 393 Pa.s, an increase of two orders of magnitude, and η* decreased with increasing frequency. The viscosity was 1.54–393 Pa.s at 0.1 Hz and decreased to 0.174–3.21 Pa.s at 10 Hz. This decrease in η* with increasing frequency was referred to as shear thinning [34]. As the frequency increased, the physical entanglement points destroyed by shear were too weak to be reconstructed, which was referred to as pseudoplastic fluid. Tanδ was the ratio of G′, G”, which reflected the state of the sample. As shown in Figure 4D, Tanδ decreased with increasing frequency, gradually changing from greater than 1 to less than 1, indicating that the rheological behavior of collagen changed from viscous to elastic properties [35]. The experimental results were similar to the experimental results of collagen from sharp nose stingray skin collagen [1], red stingray skin collagen [36], and grass carp swim bladder collagen [12]. These rheological data can provide useful information for the production of high-quality and stable collagen products.

**Figure 5:**
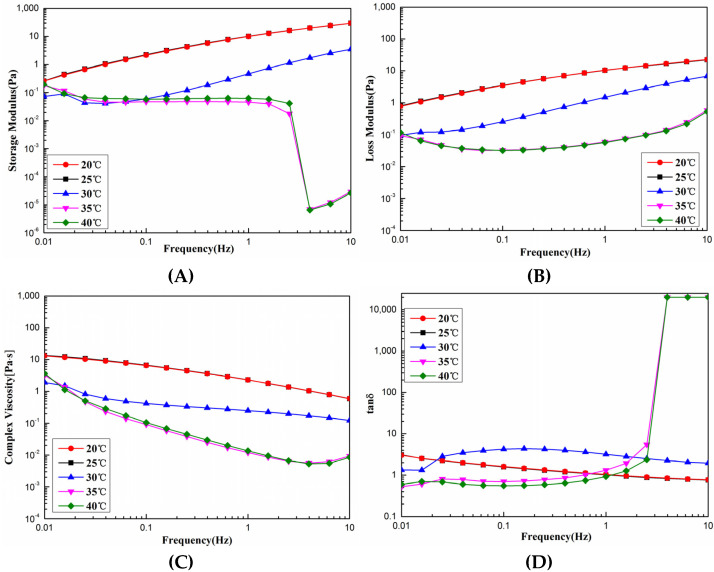
Dynamic rheological curve of SCSC at different temperatures. (**A**) Storage modulus (G′); (**B**) loss modulus (G”); (**C**) complex viscosity (η*); (**D**) tanδ.

As shown in Figure 5A,B, under the dynamic rheological curves of SCSC at different temperatures, G′ and G” were mainly related to the degree of cross-linking and the T_d_ of SCSC. When the temperature was lower than T_d_ (30.37 °C), there was very slight variation in G′ and G” with frequency. However, the temperature was higher than the T_d_ of SCSC, and the G′ value of collagen decreased rapidly when the shear frequency was higher than 1 Hz. This was caused by the fact that as the temperature increased beyond T_d_, the thermal motion energy of collagen molecules increased, the leap resistance of collagen molecular chain segments became weak, and collagen gelation occurred. As shown in Figure 5C, η* showed a decreasing trend with increasing temperature, and decreased with increasing frequency, which was related to the T_d_ and shear thinning of the sample. As the temperature exceeded the T_d_, the hydrogen bonds were broken and the triple helix structure was deconvoluted into single chains or dimers, which led to denaturing behavior of the collagen. As shown in Figure 5D, collagen exhibited elastic behavior at low frequencies and viscous behavior at high frequencies at temperatures above T_d_. These results were consistent with the properties of pseudoplastic fluids [35]. In production and processing, different production processes will cause variations in the rheological properties of SCSC during processing, with excessive viscosity adding to the difficulty of transport and processing, and too low viscosity making it difficult to set. Thus, the rheological properties of SCSC could be used to guide the production of collagen products.

### 3.7. Zeta Potential

The polar groups of the proteins tended to dissociate when the pH of the solution reached a specific value, the positive and negative ions of collagen dissociation became equal, the electrostatic charge reached zero, and the electrostatic repulsion between the molecules reached a low value and the stability was poor, where the pH value at this moment was referred to as the isoelectric point (pI) [37]. SCSC was positively charged at pH 2–6, negatively charged at pH 7–10, and the potential value was equal to 0 at a pH of 6.98 (Supplementary Materials Figure S3). The isoelectric point varied slightly among the fish species, with higher values for SCSC (6.98) compared to sea bass (6.46) [38] and yellowfin tuna (6.05) [39]. The phenomenon of this difference was likely due to the distribution of amino acid sequences and the differences among the amino acid residues [40]. 

### 3.8. Functional Properties

#### 3.8.1. Foaming Properties

**Figure 6:**
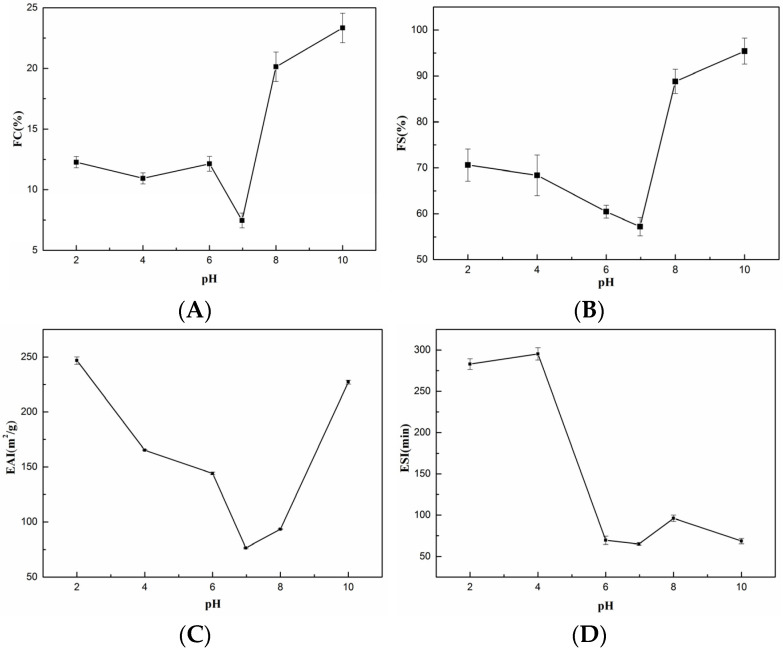
Functional properties of SCSC. (**A**) Foam capacity (FC); (**B**) foam stability (FS); (**C**) emulsifying activity index (EAI); (**D**) emulsion stability index (ESI).

Figure 6A,B shows the FC and FS of SCSC at different pH values, where FC varied from 7.46 ± 0.06% to 23.34 ± 1.22% and FS varied from 57.23 ± 1.99% to 95.39 ± 2.84% with increasing pH. FS and FC both reached their lowest points near an isoelectric point pH 6.98 (FC: 7.46 ± 0.61%, FS: 57.23 ± 1.99%). This phenomenon occurred due to the loss of electrostatic repulsion, which reduced the solubility of the collagen solution at the isoelectric point. The aggregation of undissolved proteins reduced the forces between protein and water, leading to a decrease in foaming capacity. Casein and soy protein are food foaming agents available in the market [41,42]. The FC of SCSC was superior to the FC of casein (3.95 ± 0.07%–14.25 ± 0.35%, pH = 5–9) [43], HBC 19 rice bran protein concentrate (5.2 ± 0.28%–10.03 ± 0.39%, pH = 5–9) [43] in the same pH ranges. While the FS of SCSC was higher than casein (0.17 ± 0.002–0.54 ± 0.61, pH = 5–9) [43], HBC 19 rice bran protein concentrate (3.67 ± 0,09–4.30 ± 0.16% pH = 5–9) [43], and grass carp swim bladder collagen 52.8 ± 2.45%–74.72 ± 0.71%, pH = 2–10) [12] in the same pH ranges. SCSC showed excellent foaming properties and could possibly be applied as a foaming agent in the processing of alcohol, dairy products, and other food products.

#### 3.8.2. Emulsifying Properties

Figure 6C,D shows the EAI and ESI of SCSC at different pH values, where EAI varied between 76.48 ± 0.59 m^2^g and 246.73 ± 3.37 m^2^g with increasing pH and ESI varied between 64.97 ± 1.23 min and 295.31 ± 7.46 min with increasing pH. Both ESI and EAI reached their minimum values close to the isoelectric point pH 6.98 (ESI: 64.97 ± 1.23 min, EAI: 76.48 ± 0.59 m^2^g). This phenomenon was consistent with the foaming properties, where the free radical activity in the protein diminished near the isoelectric point, which was not conducive to the emulsification properties [44,45]. Soy protein and casein are food emulsifiers available in the market [41,42]. The ESI of SCSC was better than that of chicken cartilage collagen (25.62–43.3 m^2^g, pH = 4–10) [46], peanut protein (approx. 52 m^2^g–175 m^2^g, pH = 3–7) [47], and soy protein isolate Bosa (25.94 ± 0.3–33.75 ± 1.25 m^2^g, pH = 3–8) [48] in the same pH ranges. The EAI of SCSC was superior to that of soft-shelled turtle calipash collagen (EAI < 25 min, pH = 4–10) [44], red stingray collagen (1.96 ± 0.05–80.36 ± 0.27 min, pH = 2–10), [36], peanut protein (approx. 5–100 min, pH = 3–9) [47], and soy protein isolate Bosa (16.16 ± 0.12–27.43 ± 0.86 min, pH = 3–8) [48] in the same pH ranges. The experimental results showed that SCSC might be used as supplemental collagen in food and beverages as an emulsion stabilizer [49].

#### 3.8.3. WAC and OAC

WAC refers to the amount of water absorbed and the ability of collagen to retain water, reflecting the capacity of the protein to absorb water and influence the taste and flavor of food during actual production and processing. The process of water absorption by collagen might accumulate around the collagen fibers and diffuse within the triple helix, promoting the formation of hydrogen bonds; thus, enhancing the WAC. The WAC of SCSC was 31.13 ± 0.98 g/g, which was significantly better than walnut protein isolate (3.11 ± 0.28 g/g), sunflower seed isolate (0.985 ± 0.01 g/g), and pig skin collagen (0.21 ± 0.03 g/g) [50,51,52]. The excellent WAC allowed SCSC to be applied in food and cosmetic applications. The OAC of proteins has shown to be crucial not only for the taste of the product but also for the functional properties required in the processing of meat and confectionery products. The OAC of SCSC was 26.48 ± 0.51 g/g, which is slightly lower compared to the WAC, indicating that SCSC contained more hydrophilic groups rather than hydrophobic groups. The OAC of SCSC was higher than soybean isolate (8.31 ± 0.36 g/g), green tea water soluble protein (4.5 g/g), and sorghum protein (3.39 g/g) [53,54,55]. A higher WAC with OAC could be utilized as a food stabilizer to prevent water loss in frozen products during heat treatment or thawing, as well as prevent the separation of fat fraction [56]. This showed that SCSC might have a high development value for various applications in food as well as in cosmetics.

### 3.9. Biocompatibility

The biocompatibility of the MC3T3-E1 fibroblasts was determined using the CCK-8 cell proliferation assay. The optical density (OD) value is a criterion used to determine the cell proliferation rate, where higher OD values are more favorable for cell proliferation. As shown in Table 2, the OD values on days 1, 3, and 5 of culturing were 0.521, 3.179, and 1.281, respectively. The cell proliferation rate reached the highest level on day 3 and decreased on day 5. This was possibly due to apoptosis caused by the decrease in the survival environment of the cells after high density multiplication. In general, the OD values of the cells tended to increase, where the higher cell viability values relative to the blank control indicated that SCSC facilitated cell proliferation.

**Figure 7:**
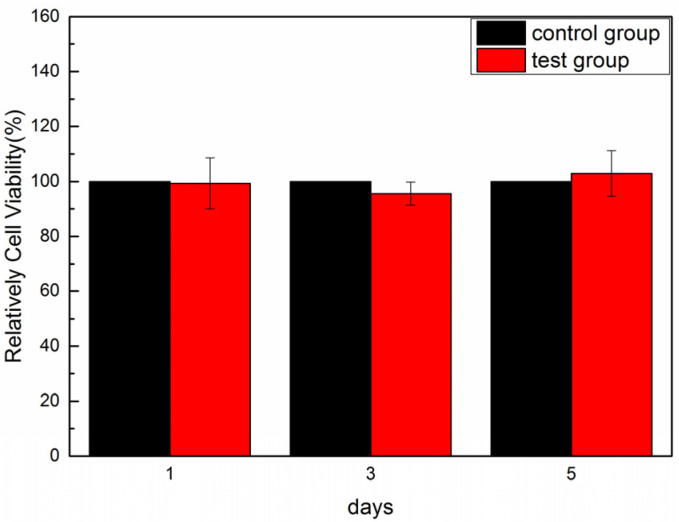
Relative cell viability of MC3T3-E1 culture on days 1, 3, and 5 in SCSC.

As shown in Figure 7, a CCK-8 assay was performed to assess the effect of different days of culturing of SCSC on the relative cell viability graph of MC3T3-E1. The relative cell viability values of the samples were greater than 95%. All the relative cell viabilities were above 70%, and according to the toxicity grade assessment of IOS, the toxicity grade of all samples with a cell viability greater than 70% was 0 [57]. These results were similar to those of grass carp swim bladder collagen [12] and red stingray skin collagen [36], indicating that SCSC is non-cytotoxic and biocompatible. Thus, SCSC has great potential for application in biomedical materials.

**Figure 8:**
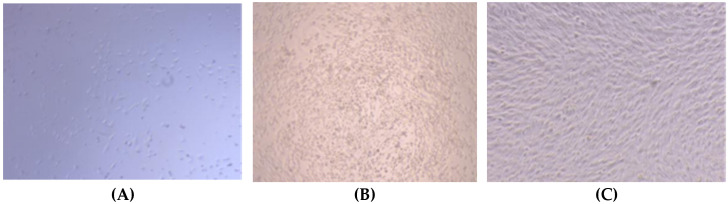
Cell morphology of MC3T3-E1 in SCSC at different days of culture. (**A**) Culture for 1 day; (**B**) culture for 3 days; (**C**) culture for 5 days.

As shown in Figure 8, the morphology of the cells cultured with SCSC was observed using an inverted microscope (ECLIPSE Ti, Nikon, Japan). On the first day, the MC3T3-E1 cells were sparse, and a shuttle-shaped cell morphology was observed. On the third day, the number of shuttle-shaped cells increased significantly. On the fifth day, the cells were stacked together in a squamous pattern, with no cell lysis or morphological abnormalities observed. These results were consistent with the conclusion of cell proliferation, where SCSC could promote the growth of MC3T3-E1 cells, indicating that SCSC was biocompatible and was a potential biomaterial with great development value.

## 4. Conclusions

In this work, SCSC was extracted from the skin of silver carp and identified as type I collagen using electrophoretic experiments and spectroscopic analysis, with a triple helix structure that was intact. The amino acid sequence analysis identified SCSC as *Hypophthalmichthys molitrix*. The T_d_ tests showed that the T_d_ value of SCSC was higher than deep-sea fish sources such as Pacific cod, Sharpnose, and Arabian mackerel. The Td value of SCSC was also better than that of some freshwater fish with similar survival environments, such as sturgeon and Mozambique tilapia. Interestingly, the T_d_ of SCSC was higher than that of human placental collagen. Functional property analyses indicated better functional properties of SCSC compared to other proteins from terrestrial sources in terms of WAC, OAC, foaming properties, and emulsifying properties. Rheological property analyses indicated that SCSC had shear thinning properties and was a pseudoplastic fluid, while the biocompatibility results indicated that the MC3T3-E1 cells could proliferate and maintain normal morphology in the SCSC solutions. Thus, SCSC could be applied as an alternative collagen resource on the market. The collagen extracted from the by-products of silver carp could greatly enhance the economic value of silver carp and realize the high-value utilization of low-value products.

## Figures and Tables

**Table 1:** Matching peptide coverage of SCSC amino acid sequences.

GI	Score	Mass/pi	Sequences	Coverage ^1^	Protein Description
AIL02135.1	847	138,365/5.44	19	20%	*Hypophthalmichthys molitrix α_1_* (I)
AUF74474.1	2009	127,755/9.36	31	26%	*Hypophthalmichthys molitrix α_2_* (I)

^1^ Matched peptide coverage of the protein sequence.

**Table 2:** Classification of OD relative cell viability and toxicity of SCSC at different days of culture.

Time (D)	OD Value	Relative Cell Viability (%)	Classification of Toxicity
1	0.521	99.30 ± 9.3	0
3	3.179	95.54 ± 4.2	0
5	1.281	102.91 ± 8.33	0

## Data Availability

Data is contained within the article and Supplementary Materials.

## Supplementary Materials

The following supporting information can be downloaded at: https://www.mdpi.com/article/10.3390/foods11192985/s1, Figure S1: Amino acid sequence of *Hypophthalmichthys molitrix* type I collagen subunit α1. Bold red peptides: Matched peptide in the NCBI database; Figure S2: Amino acid sequence of *Hypophthalmichthys molitrix* type I collagen subunit α2; Bold red peptides: Matched peptide in the NCBI database. Figure S3: Zeta potential of SCSC.
