# Supplementary material for: Valorization of Fish Processing By-Products: Microstructural, Rheological, Functional, and Properties of Silver Carp Skin Type I Collagen
Source: Foods. 2022 Sep 24;11(19):2985. doi: 10.3390/foods11192985 (PMC9562877; doi:10.3390/foods11192985)
Supplement: Supplementary file 1 [file foods-11-02985-s001.zip › foods-1929216-supplementary.pdf]

Valorization of fish processing by-products: microstructural, rheological, functional, and properties of silver carp skin type I collagen

Yongxin Guan[1,2], Jianlin He[2], Junde Chen[2,*], Yushuang Li[2], Xingkun Zhang[2], Yan Zheng[2], Linyan Jia[1,*]

1 College of Chemistry and Chemical Engineering, Mudanjiang normal University, Mudanjiang 157011, China
2 Technical Innovation Center for Utilization of Marine Biological Resources, Third Institute of Oceanography, Ministry of Natural Resources, Xiamen 361005, China

* Correspondence: jdchen@tio.org.cn (J.C.); 1002002@mdjnu.edu.cn (L.J.);
Tel./Fax: +86-592-215527 (J.C.); +86-453-6426868 (L.J.)

Supplementary Materials

Figure S1. Amino acid sequence of *Hypophthalmichthys molitrix* type I collagen subunit α1. Bold red peptides: Matched peptide in the NCBI database.
Figure S2. Amino acid sequence of *Hypophthalmichthys molitrix* type I collagen subunit α2. Bold red peptides: Matched peptide in the NCBI database.
Figure S3. Zeta potential of SCSC.

1	MFSFVDIRLA LLLSATVLLA RGQGEDDRTG GSCTLDGQVY NDRDVWKPEP
51	CQICVCDSGT VMCDEVICED TTDCPNPVIP HDECCPVCPD DDFQEPSVEG
101	PRGTPGEKGD RGPPGPPGND GIPGQPGLPG PPGPPGPPGL GGNFSPQMSG
151	GFDEK**SGGAM AVPGPMGPMG PR**GPPGPPGT PGPQGFTGPP GEPGEAGAPG
201	PMGPRGAAGP PGKNGEDGES GKPGRPGERG PPGPQGARGF PGTPGLPGIK
251	GHR**GFSGLDG AKGDTGPSGP K**GEAGAPGEN GTPGAMGPRG LPGERGRAGP
301	PGAAGARGND GAAGAAGPPG PTGPAGPPGF PGGPGAK**GEV GPQGAR**GAEG
351	PQGARGEAGN PGPAGPAGPA GNNGADGAAG PKGSPGTPGI AGAPGFPGPR
401	GPPGPSGAAG APGPKGNTGE VGAPGAKGEA GAKGEAGAQG VQGPPGPPGE
451	EGKRGARGEP GAAGGR**GPPG ERGAPGAR**GF PGADGSAGPK GAPGERGGPG
501	VVGPK**GATGE PGRNGEPGMP GSKGMTGSPG SPGPDGKTGP SGTPGQDGRP**
551	**GPPGPVGAR**G QPGVMGFPGP KGAAGEAGKP GER**GVMGAVG ATGAPGK**DGD
601	VGAPGAPGPA GPAGERGEQG PAGPPGFQGL PGPQGATGEP GKSGEQGLPG
651	EAGAPGPSGS RGDRGFPGER GAPGPAGPAG ARGSPGSAGN DGAKGDSGAP
701	GAPGAQGPPG LQGMPGERGA AGLPGLKGDR GDQGAK**GTDG APGKDGIR**GM
751	TGPIGPPGPA GAPGDKGETG APGLVGPAGA RGPPGERGET GAPGPAGFAG
801	PPGADGLPGA K**GEAGDNGAK GDAGPPGPSG ATGAPGPQGP VGATGPK**GAR
851	GAAGPPGATG FPGAAGRVGP PGPAGNAGPP GPPGPAGKEG QKGSRGETGP
901	AGRTGEIGTP GPPGAPGEK**G TPGAEGPTGP SGTPGPQGIN GQRGIVGLPG**
951	**QR**GERGFPGL PGPSGEPGK**Q GPSGPSGERG PPGPMGPPGL AGPPGEPGRE**
1001	**GTPGNEGSAG R**DGAPGPKGD RGETGAAGTP GAPGPPGAPG PIGPAGK**TGD**
1051	**RGESGPAGPS GAVGLTGPRG PVGPAGAR**GD KGETGEAGER GMKGHRGFTG
1101	IQGPPGPPGP SGEPGPAGAS GPAGPRGPAG SSGPAGK**DGM SGLPGPIGPP**
1151	**GPR**GRNGEIG PAGPPGAPGP PGPPGPSGGG FDIGFIAQPQ EKAPDPFRHF
1201	RADDANVMRD RDLEVDTTLK SLSQQIESIM SPDGTKKNPA RTCRDLKMCH
1251	PDWKSGEYWI DPDQGCNQDA IKVYCNMETG ETCVYPTEST IPKKNWYTSK
1301	NIKEKKHVWF GEAMTDGFQF EYGSEGSKAE DVNIQLTFLR LMSTEASQNI
1351	TYHCKNSIAY MDQASGNLKK ALLLQGSNEI EIRAEGNSRF TYSVTEDGCT
1401	SHTGAWGKTV IDYKTTKTSR LPIIDIAPMD VGAPNQEFGI EVGPVCFL

Figure S1. Amino acid sequence of Hypophthalmichthys molitrix type I collagen subunit α1. Bold red peptides: Matched peptide in the NCBI database.

1 MLSFVDTRIL LLLAVTSYLA SCQSSTRGPK GPRGERGPKG PDGKPGKPGL

51 PGPPGPPGPP GLGGNFAAQY DGAK**GIEAGP GPMGLMGPR**G PSGPPGAPGP

101 QGFQGHAGEP GEPGQAGAVG SRGPPGPPGK NGEDGNNGRP GKPGDRGAPG

151 AQGARGFPGT PGLPGMKGHR **GYTGLDGRK**G EPGAAGAKGE NGAPGSNGTP

201 GQRGGRGLPG ERGR**VGPSGP AGAR**GADGNI GPAGPAGPLG AAGLPGFPGA

251 PGPK**GEIGPA GPTGPSGPQG QR**GEPGTNGA VGPAGPPGNP GANGINGAKG

301 AAGPPGVAGA PGFPGPRGGP GPQGPSGASG PR**GLAGDPGP VGVKGDSGVK**

351 **GEPGSAGPQG PPGPSGEEGK RGSTGEQGST GPLGMR**GPRG AAGTRGLPGL

401 AGR**SGPMGMP GAR**GATGAPG ARGPPGDAGR **AGEPGLVGAR** GLPGSPGSSG

451 PPGK**EGPAGP AGQDGRSGPP GPTGPR**GQPG NIGFPGPKGP SGEPGKPGEK

501 **GPTGPTGLR**G QPGPDGNNGP PGPVGLAGAP GEKGEQGPSG APGFQGLPGP

551 AGPAGEAGKS GDR**GIPGDQG AQGPAGVKGE RGNPGPAGAA GAQGPIGARG**

601 **PAGTPGPDGN KGEPGAVGAA GAAGHQGAAG MPGER**GAAGT PGPKGEKGEQ

651 GYR**GLEGNAG R**DGARGAPGP SGPPGPAGAN GDKGETGSFG PPGPAGARGA

701 PGER**GESGPA GPSGFAGPPG ADGQTGQR**GE KGPAGVKGDA GPPGPAGPAG

751 NTGPLGPSGP VGPPGARGDS GPPGLTGFPG AAGRVGPPGP SGIVGPAGPT

801 GAPGKDGPRG AR**GDVGPAGP PGENGMMGPP GLAGEK**GSPG ESGAPGAPGP

851 AGPQGQLGSQ GFNGLPGSRG DRGLPGGPGA VGDAGRVGPA GAPGAR**GPAG**

901 **NIGMPGMTGP QGEAGREGSP GNDGPPGRPG AAGLKGDR**GE PGSPGAAGPV

951 GAPGPNGPSG AVGRPGNR**GE SGPSGSSGPV GPAGARGAPG PAGPR**GEKGV

1001 AGDKGERGMK GLRGHPGLQG MPGPNGPSGD SGPAGIAGPA GPR**GPAGPNG**

1051 **PPGK**DGSNGM PGAIGPPGHR GPPGYVGPAG PPGSPGLPGP PGQAGGGYDT

1101 SGGYDEYRAD QASLRAKDYE VDATVKSLNT QIENLLSPEG SKKNPARTCR

1151 DIRLSHPEWS SGFYWIDPNQ GCTMDAIKAY CDFSTGQTCI HPHPESIPQK

1201 NWYKSSQEKK HIWFGETING GTEFSYNDET LSPQSMATQL AFMRLLANQA

1251 VQNITYHCKN SIAYMDAENG NLKKAVLLQG SNDVELRAEG NSRFTFSVLE

1301 DGCSRHTGQW GKTVIEYRTN KPSRLPILDI APLDIGGADQ EFGLDIGPVC

1351 FK

Figure S2. Amino acid sequence of Hypophthalmichthys molitrix type I collagen subunit α2. Bold red peptides: Matched peptide in the NCBI database.

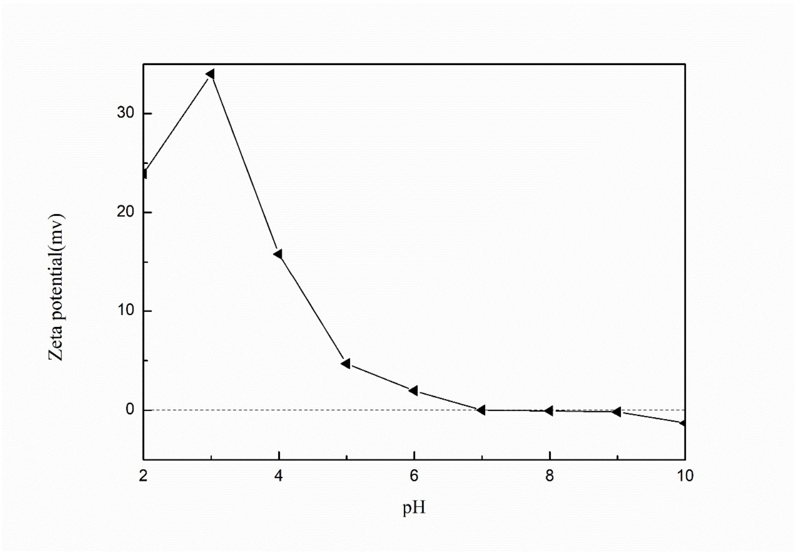

Figure S3. Zeta potential of SCSC.
